# Chondromyxoid Fibroma of the Distal Tibia: A Rare Case Report

**DOI:** 10.7759/cureus.51319

**Published:** 2023-12-30

**Authors:** Ousama Jelti, Oussama El Alaoui, Adnane Lachkar, Najib Abdeljaouad, Hicham Yacoubi

**Affiliations:** 1 Department of Orthopedics and Traumatology, Mohammed VI University Hospital, Oujda, MAR; 2 Faculty of Medicine and Pharmacy, Mohammed Ist University, Oujda, MAR; 3 Department of Orthopedics and Traumatology, Centre Hospitalier Universitaire (CHU) Mohammed VI Oujda, Oujda, MAR

**Keywords:** case report., surgery, tibia, benign osseous tumor, chondromyxoid fibroma

## Abstract

Chondromyxoid fibroma is one of the rarest bone tumours, occurring most frequently in adult men in their second and third decades. It generally affects the metaphysis of long bones, particularly the femur and tibia. Diagnosis can pose differential challenges with various tumor types, particularly chondrosarcoma, requiring separate management. We present a case of chondromyxoid fibroma of the distal tibia detected by soft tissue swelling. Clinical, epidemiological and radiological aspects will be discussed.

## Introduction

The initial description of chondromyxoid fibroma (CMF) dates back to 1943, with the work of Jaffe and Lichtenstein [1]. Around 500 CMF cases have been described in the entire literature [2]. This benign bone tumor, characterized by slow growth resulting from chondroblastic derivation [3-6], typically manifests during the second and third decades of life, showing a preference for the metaphyseal region of long bones, particularly in the lower limbs. In approximately 50% of cases, the proximal tibia is affected, followed by the distal femur, pelvis, metatarsals, and calcaneus. Due to the presence of cells exhibiting pleomorphism, these tumors can be mistaken for chondrosarcoma. However, they represent less than 1% of primary bone tumors [7]. Periosteal chondromyxoid fibroma has been identified with impression cytology as a confirmatory method [8]. Our presentation involves a case of CMF localized in the distal tibia, setting it apart from the more common presentations observed in the proximal tibia.

## Case presentation

**Figure 1:**
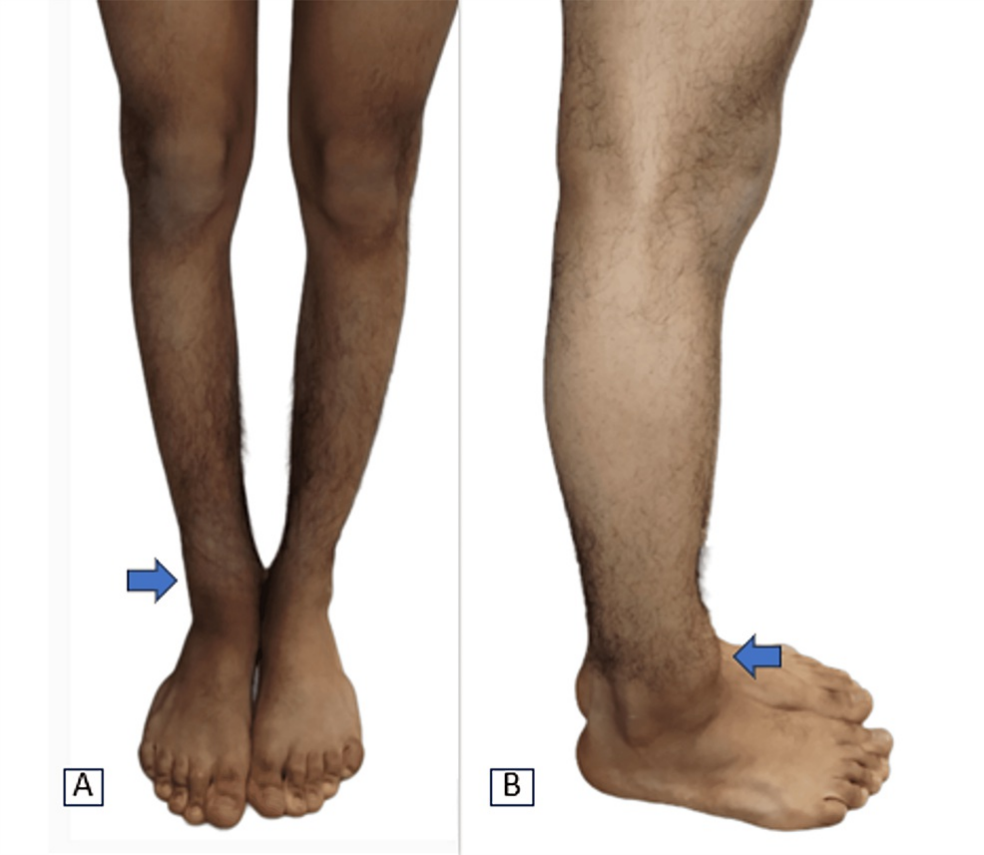
Clinical picture of tumor (A, B).

We describe the case of a 17-year-old adolescent who consulted us for a painful swelling above his right ankle, present for two years and progressively increasing in size. On physical examination, the patient presented with a hard, fixed and tender bony swelling on the distal part of the right tibia. The lesion was not adherent to the skin and was not associated with ulceration or elevated temperature (Figure 1).

**Figure 2:**
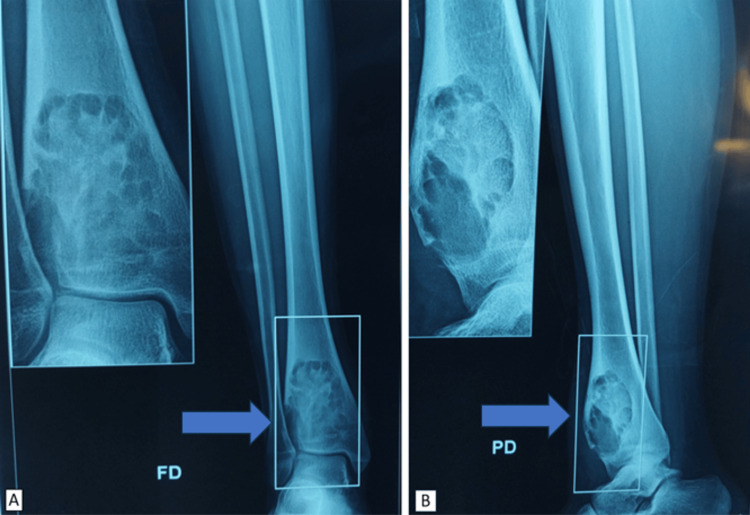
Anteroposterior (A) and lateral (B) standard X-ray showing lytic remodelling of the tibial pilon, with cortical blowout.

The swelling was diffuse and extensive. Neurovascular status of the right lower limb was normal. The patient had no other symptoms, and the systemic physical examination revealed no abnormal findings. No medical, family, allergic, drug or social history was relevant. Laboratory investigations, including blood count, erythrocyte sedimentation rate (ESR) and C-reactive protein (CRP), were within normal limits. Conventional radiographs of the right ankle and tibia in anteroposterior and lateral projection revealed a lytic image at the level of the tibial pilon, presenting a polylobed aspect of type IA according to Lodwick's classification (Figure 2).

**Figure 3:**
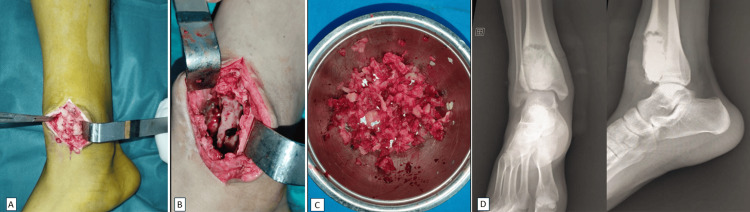
Intraoperative images showing tumor resection with cement filling (A-C). Post-operative radiological control (D).

The patient underwent a surgical procedure involving en bloc biopsy-exeresis of the tumor with the interposition of surgical cement (Figure 3).

**Figure 4:**
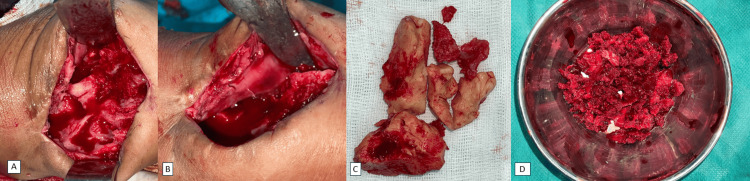
Intraoperative images showing cement removal (A-C), with placement of cancellous bone graft (D).

After four months, the cement was replaced by a cancellous bone graft (Figure 4).

**Figure 5:**
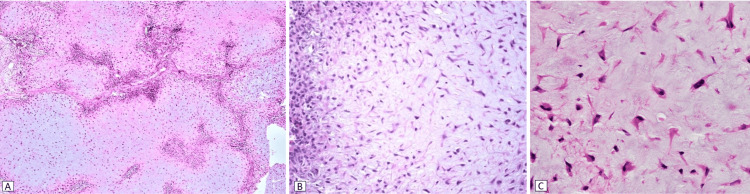
Microphotographs of the lesion reveal a lobular architecture (A), featuring hypercellularity at both the center and periphery (B), with the presence of stellate cells at the center of the proliferation (C).

The histological study confirmed the diagnosis and revealed a benign tumor proliferation of lobulated architecture, with hypercellular central zones, made up of stellate cells with ovoid to fusiform nuclei, devoid of cytonuclear atypia, and surrounded by abundant eosinophilic cytoplasm, presenting bipolar or multipolar cytoplasmic extensions. The latter are arranged within a myxoid background. The periphery of the lobule is hypercellular, with spindle-shaped cells, non-atypical ovoid nuclei and abundant cytoplasm. No malignancy (Figure 5).

Postoperatively, the patient was placed in partial weight-bearing for an initial three weeks, then resumed full weight-bearing after five weeks. At the last follow-up visit, 12 months after surgery, the patient was asymptomatic and showed no recurrence.

## Discussion

Chondromyxoid fibroma, a benign bone neoplasm, remains a rarity, accounting for less than 0.5% of all bone tumors and a mere 2% of benign tumors. Initially characterized by Jaffe and Lichtenstein [1], CMF exhibits variable proportions of cartilaginous and fibromyxoid tissues. It predominantly afflicts adults in their middle years, around the age of 30, although it can also manifest in children and the elderly [9,10]. Notably, there is a male predominance, with a sex ratio of 2:1. The most common location is in the metaphyseal region of the long bones, with the proximal tibia the most frequently affected site, followed by the distal femur, pelvis and foot. Long bones are involved in younger patients, while flat bone involvement occurs more frequently in older patients [11].

Clinical manifestation usually takes the form of chronic pain (85%), swelling (65%), restriction of movement and, more rarely, pathological fracture [12-14]. With regard to these aspects, our case shows notable similarities with previous publications, particularly with regard to the presence of pain and swelling. The average duration of pain is around 22 months, while that of swelling is around 10 months [13,14]. Our case demonstrates significant concordance with the literature in this context.

Imaging is of particular interest in establishing a positive diagnosis; indeed, the characteristic radiological appearance takes the form of a lacunar, osteolytic, metaphyseal, eccentric image, with a long axis parallel to the axis of the bearing bone and containing fine intratumoral septa. This pictorial presentation is surrounded by a fine border of sclerosis, suggesting a benign lesion. The cortex is frequently blown and thinned into a shell. Cases of cortical effraction have been reported [9,10,12]. Intratumoral calcifications are exceptional, occurring in only 1.5% to 3% of cases according to documented series [9]. Cross-sectional imaging modalities such as computed tomography (CT) and magnetic resonance imaging (MRI) do not allow a positive diagnosis, although MRI remains a sensitive technique for the detection of bone tumors. The MRI appearance of chondromyxoid fibromas varies according to the proportions of cartilaginous, myxoid and fibrous tissue. Myxoid areas show hyposignal in T1 and hypersignal in T2. As for the fibrous component, signal intensity varies according to the degree of vascularization [12].

Overall, the tumor exhibits a firm consistency, a grayish-white hue and may have a lobulated or pseudolobulated configuration. Its structure is reminiscent of fibrous tissue or hyaline cartilage, similar to other tumors such as chondroblastoma or chondrosarcoma. Lesions often lead to thinning of the cortex and rarely to destruction of the trabecular bone. Occasionally, the tumor may present areas of hemorrhagic and cystic degeneration, sometimes mimicking aneurysmal bone cysts. Many chondromyxoid fibroids CMF display morphological features corresponding to different stages of chondrogenesis [15,16].

Low-grade chondrosarcoma can sometimes present a histological similarity with CMF, except for the absence of myxoid elements and soft tissue involvement. Chondrosarcoma has distinct demographic and radiographic characteristics. The peak incidence of chondrosarcoma occurs around the sixth and seventh decades of life, whereas CMF occurs in the second and third decades of life. Radiographically, chondrosarcoma is centrally located and shows abundant calcifications. Chondrosarcoma and CMF sometimes show cortical expansion. In old-type or higher-grade chondrosarcomas, soft tissue involvement and cortical erosion are observed [17,18].

Treatment of CMF is primarily surgical, involving extensive resection of tumor tissue, followed by filling of the residual cavity with cancellous or corticospongiosa. Simple curettage exposes the patient to the risk of recurrence. Radiotherapy may be considered as an option for unresectable tumors [12]. Recurrence usually occurs within two years, although cases of recurrence have been reported up to 19 years after initial treatment [17, 19-21]. Thus, regular follow-up of patients, including periodic physical and radiological examinations for at least two years, is recommended [11,22].

## Conclusions

Chondromyxoid fibroma (CMF) is a rare bone tumor composed of chondroid, myxoid and fibrous elements, often confused with other entities with similar radiological and pathological features, frequently leading to misdiagnosis. Diagnosis of this tumor relies on imaging and histological examination, although cytogenetics and immunohistochemistry can provide additional information. It is essential to distinguish CMF from malignant tumors such as chondrosarcoma. Therapeutic management of this tumor involves wide excision of the lesion in healthy areas, which is the only way to prevent local recurrence.
